# Editorial: Tau Pathology in Neurological Disorders

**DOI:** 10.3389/fneur.2021.754669

**Published:** 2021-09-24

**Authors:** Sonia Do Carmo, Maria Grazia Spillantini, A. Claudio Cuello

**Affiliations:** ^1^Department of Pharmacology and Therapeutics, McGill University, Montreal, QC, Canada; ^2^Department of Clinical Neurosciences, Clifford Allbutt Building, University of Cambridge, Cambridge, United Kingdom; ^3^Department of Neurology and Neurosurgery, McGill University, Montreal, QC, Canada; ^4^Department of Anatomy and Cell Biology, McGill University, Montreal, QC, Canada; ^5^Department of Pharmacology, Oxford University, Oxford, United Kingdom

**Keywords:** tau pathology, tauopathies, Alzheimer's disease, neurodegeneration, dementia, tau therapeutics

The histological alterations now known as neurofibrillary tangles (NFTs) were first described in 1906 by Alois Alzheimer using light microscopy and silver staining (1); in the late 1960's it was shown by electron microscopy that they were made of paired helical filaments (PHFs) and a small percentage of straight filaments (SFs) (2, 3). Around this time, it was also shown that abundant NFTs were associated with cognitive decline (4, 5). Between 1985 and 1991 it was shown by immunohistochemistry, biochemical approaches and molecular cloning that PHFs and SFs are made of hyperphosphorylated tau protein (6–11) a microtubule-associated protein first described in 1975 (12). Tau filaments from Alzheimer's disease were shown to be made of a structured core and the less structured fuzzy coat (10, 13, 14). These pioneering accomplishments paved the way to further recognize the complexity of tau translation and splicing leading to the identification of the different tau isoforms in the healthy brain and modification such as phosphorylation, truncation and conformational changes in neurodegenerative diseases (7, 15–18). In normal human brain, six tau isoforms are expressed from a single gene by alternative mRNA splicing (19, 20). They differ by the presence or absence of two amino-terminal inserts and an extra repeat of 31 amino acids in the C-terminal region. Depending on its presence, one can divide tau isoforms into two groups, three isoforms with three repeats and three isoforms with four repeats. Both groups of isoforms are expressed at similar levels in normal brain. The repeats and some adjoining sequences make up the microtubule-binding region of tau. They also form part of the filament core, suggesting that physiological function and pathological assembly are mutually incompatible. In Alzheimer's disease, all six tau isoforms are present in PHFs and SFs (15). Electron microscopy and image reconstruction showed that tau filaments from Alzheimer's disease are made of two identical C-shaped protofilaments (21). However, the resolution was insufficient to see individual amino acids. This changed in 2017, when the structure of the Alzheimer tau fold was obtained at near-atomic resolution using electron cryo-microscopy (cryo-EM) (22). The same tau fold has been described in multiple individuals with Alzheimer's disease (23), as well as in some cases with prion protein amyloidosis (24) and in primary age-related tauopathy (PART) (25). Different tau folds have been described in Pick's disease (23), chronic traumatic encephalopathy (26), and corticobasal degeneration (27).

These findings tell us that Alzheimer NFTs are made of filamentous tau, but they do not say anything about the relevance of tau assembly for the disease process. This required human genetics. In June 1998, three publications reported mutations in *MAPT*, the tau gene, in familial forms of frontotemporal dementia (28–30). Even though *MAPT* mutations do not cause Alzheimer's disease, these findings proved that dysfunction of tau protein is sufficient to cause neurodegeneration and dementia. To date, 65 missense, deletion and intronic mutations in *MAPT* have been shown to cause disease. Gene dosage mutations have also been reported (31). Importantly, filamentous tau assemblies were present in all cases. Depending on the mutations, assemblies are made of either 3R, 4R, or 3R+4R tau. Similar findings have been described for idiopathic human tauopathies. Thus, Pick's disease is a 3R tauopathy, whereas progressive supranuclear palsy, globular glial tauopathy, corticobasal degeneration, and argyrophilic grain disease are 4R tauopathies. Alzheimer's disease, familial British dementia, familial Danish dementia, chronic traumatic encephalopathy and Guam-Parkinsonism-Dementia are 3R+4R tauopathies.

In 2009, it was shown that assembled tau exhibits prion-like properties in experimental systems (32, 33). It is now well-established that a tau seed can induce the assembly of monomeric protein, but it remains to be established what role the prion-like spreading of assembled tau plays in human brain. Although the staging of tau pathology is associated with development of dementia (34–37) and is consistent with prion-like spreading, it cannot prove its existence. Perhaps the best evidence for prion-like spreading in human brain comes from a study showing the absence of assembled tau in denervated frontal cortex from Alzheimer's disease (38). Using sucrose gradient fractionation of the brains from mice transgenic for human mutant P301S tau and a cellular assay, it has been shown that small tau filaments are the most potent seeds (39). It remains to be determined which species of assembled tau cause neurodegeneration. It has been suggested that tau oligomers may play an important role (40–42). Current knowledge also suggests that the intercellular transfer of tau aggregates is dependent on the tau species (43) and is likely mediated through mechanisms such as the release of tau seeds into the extracellular space in a free form or within vesicles, such as exosomes, which enter recipient neighboring cells by fluid-phase or receptor-mediated endocytosis or by vesicle fusion. The transfer of tau seeds to recipient cells can also occur through nanotubes [reviewed in (44)]. The recipient cells can be either neurons or other brain cell types such as microglia, astrocytes, oligodendrocytes and endothelial cells as highlighted in this Research Topic.

Notwithstanding that the presence and importance of tau and its modifications in neuropathology were first studied in Alzheimer's disease, tau inclusions are also represented in other neurodegenerative diseases grouped under the umbrella term “tauopathies.” The strong association between NFTs and cognitive capabilities in the continuum of Alzheimer's disease, and observed also in Down syndrome, was described in a wealth of literature (34, 45–57). These observations were also corroborated by the recent report of an autosomal dominant Alzheimer's disease case who presented mild cognitive impairment at the age of 70, 3 decades later of what expected in the family and who presented with extreme amyloid burden but minimal tau pathology (58). In addition to Alzheimer's disease, tau pathology has been described in other neurodegenerative diseases which lack significant Aβ pathology including Pick's disease, progressive supranuclear palsy, corticobasal degeneration, and frontotemporal dementia and parkinsonism linked to chromosome 17, among others (30, 59–62) [reviewed in (63, 64)]. In consequence the term “tauopathy” used to describe the pathology in a family with MAPT mutation (61) encompassing now all the above neurodegenerative diseases.

Still, despite the colossal amount of knowledge on tau biology gathered so far, significant questions related to how tau transitions from the homeostatic into the disease state remain at least partially unanswered: (1) How do the differential levels of tau post-translational modifications and aggregation affect tau propagation and its biological activity? (2) What is the contribution of tau regions outside the microtubule binding domain to tau physiology and pathology? (3) Which factors define the phenotypic heterogeneity of tauopathies; is miRNA-mediated regulation of tau involved? (4) What are the functional consequences of tau interaction with cellular organelles such as the nucleus and mitochondria? (5) How and how much does the transmission of tau to non-neuronal cells contribute to tauopathy progression and what is the functional consequence of such transmission in the recipient cells? (6) What is the contribution of tau pathology to the malfunction of the brain vasculature and neurovascular unit in Alzheimer's disease? (7) To which extent does tau intersect with other pathologies to drive neurodegeneration and cognitive dysfunction? (8) How does the current knowledge of tau biology translate into tau-targeted therapies, and can these be of benefit in tauopathies other than Alzheimer's?

We present this Research Topic and e-book to provide an insight into some of these questions and highlights the most recent advances in understanding the molecular and cellular mechanisms underlying the evolution of tau pathology in Alzheimer's disease and in other tauopathies.

In this Research Topic, the extent of tau post-translational modifications, the assembly states and conformations of tau, the distribution of tau in different cell types, the spreading of tau to cells and tissues and the toxic effects of abnormal tau are comprehensively reviewed by Kang et al. who conclude that “although heterogeneity [of tau biology] in the here and now is an inconvenient truth, embracing this effect, defining its origins and then adjusting approaches may pave the way for more sophisticated testing and more realistic interventions.”

The extent of tau post-translational modifications across tauopathies, their frequency, and their effect on tau function, aggregation and degradation are further reviewed by Alquezar et al. It is noteworthy that the vast number of sites on the tau protein that can be targeted by specific modifications, often on the same amino acid residue, opens the door for a competition between post-translational modifications that defines a dynamic code regulating tau function, aggregation and degradation and could therefore explain the heterogeneity of tauopathies. The significance of tau phosphorylation is further emphasized by Duquette et al. who discuss that the pattern of tau hyperphosphorylation varies greatly in physiological and pathological conditions. Tau hyperphosphorylation appears mainly protective and reversible in brain development and in hibernating animals. However, in pathological conditions the pattern of tau hyperphosphorylation is disease-specific in the same way as the atomic structure of their intrinsic tau filaments as revealed by cryo-EM.

The disease-specificity of tau phosphorylation is further highlighted in amyotrophic lateral sclerosis (ALS) and in ALS frontotemporal spectrum disorder (ALS-FTSD) as reviewed by Strong et al. where the distinctive pathological phosphorylation at Thr175 promotes exposure of the phosphatase activating domain in the tau N-terminus thereby activating GSK3β-mediated phosphorylation at Thr231 leading to the formation of tau oligomers. Indeed, it has been shown that tau phosphorylation and aggregation are intimately linked and tau hyperphosphorylation by proline-directed stress kinases, such as GSK3β favors tau oligomerization (65). Novel information regarding the reason for which oligomeric tau might be more neurotoxic than fibrillar tau is provided by Jiang et al. Using a seeding assay in primary neuron cultures expressing human 4R0N tau Jiang et al. demonstrate that oligomeric seeds of tau show a greater co-localization with RNA binding proteins associated with stress granules, an element that could contribute to increased pathology.

The molecular organization of the tau protein in relation to the functional interactions of diverse tau regions, including the particular features of the tau non-microtubule binding region are eloquently reviewed by Brandt et al. Of relevance, tau knockout in mouse models results in a several of effects, with only some of them related to microtubule-dependent processes. This highlights the role of the N-terminal region of tau in the organization and function of membrane organelles, such as the plasma membrane (66, 67) and synaptic vesicles as well as its involvement in tauopathies.

Tau aggregates are mostly found within neurons where they disrupt synaptic plasticity, cell signaling, and DNA integrity eventually leading to cell death. Indeed, stress and pathological conditions promote tau accumulation in cellular compartments other than axons which are considered their main sub-cellular localisation. As such, tau can also be found in the soma, the dendrites, and the nucleus where it can interact with nuclear components. As reviewed by Diez and Wegmann, the dual localisation of tau in cytoplasm and nucleus suggests the transport of tau between the two compartments by mechanisms still to be defined given the absence of nuclear localisation signals. The nuclear tau interaction with DNA appears to be regulated by its phosphorylation and it has been suggested as protective of double stranded DNA breaks and participant of regulation of gene expression.

Such nuclear localization may also promote chromosome stability. On the other hand, cytoplasmic tau could alter the structure of the nuclear envelope and promote the aggregation of nucleoporins in the cytoplasm. This would alter the nucleocytoplasmic protein transport and therefore impacting major cellular signaling pathways. In response to stress nuclear tau can also translocate to the nucleolus and be found in stress granules in the cytoplasm as revealed in ALS and ALS-FTSD and discussed by Strong et al. Moreover, tau pathology impacts negatively adult hippocampal neurogenesis in both Alzheimer's disease and tau mouse models as summarized by Houben et al. This provides a new insight on a less documented tau-related pathological pathway leading to cognitive deficits. However, the studies carried so far have produced conflicting results.

The presence of tau aggregates is being increasingly reported in non-neuronal brain cells. Whether such aggregates derive directly from the intracellular microtubule tau detachment of glial expressed tau or from the glial uptake of neuronal tau is still a matter of debate (68–70). It is well-established that astrocytes play a critical role in supporting neuronal function and that pathological astrocytic changes are associated with and precede neuronal loss in Alzheimer's disease and overt tau aggregation in mouse models of tau aggregation (71). Interestingly, while tau deposition in astrocytes is mostly seen in aging, it can also be found in tauopathies, although with varying frequency according with the disease condition, as reviewed by Reid et al. This deposition of tau impacts the function of astrocytes through mechanisms which includes disruption of calcium signaling, gliotransmitters release, and immune responses consequentially affecting neuronal function. In addition, astrocytes may participate in the spreading of tau *via* the astrocytic water channel aquaporin-4 (AQP4) involved in the elimination of aberrant proteins.

The disruption of astrocytic and microglial function by tau may also participate in vascular dysfunction, an early characteristic of Alzheimer's disease (72). Canepa and Fossati discuss studies *in vitro*, in animal and human supporting the view that tau has deleterious effects on cellular and molecular pathways in vascular and immune cells which lead to the dysregulation of the neurovascular unit. These authors also propose mechanisms mediating the effects of tau on the cerebrovasculature, including tau-induced mitochondrial dysfunction which would lead to increased reactive oxygen species and decreased ATP production and caspase activation. Further to it, Bryant et al. provide novel data showing that microvessels isolated from the dorsolateral prefrontal cortex of Alzheimer's subjects display extensive tau pathology (Braak V/VI, B3) and a strong upregulation of endothelial senescence and leukocyte adhesion-related genes.

The disease-specificity of tau accumulation in glia and the role of oligodendrocytes in tau spreading is further highlighted by Zareba-Paslawska et al. Using a humanized tau mouse line overexpressing all six human tau isoforms in a murine tau knockout background inoculated with insoluble tau extracts from corticobasal degeneration brain homogenates, they show a 4R-tau dependent spreading primarily mediated by oligodendrocytes.

Given the critical role of tau in Alzheimer's and other tauopathies, understanding how tau expression, splicing, post-translational modifications and aggregation are regulated is essential. Boscher et al. describe the involvement of microRNAs (miRNAs) in such regulation. In particular, loss of the miR-132/212 cluster is strongly associated with memory decline and increased tau pathology. Intriguingly, Boscher et al. show that deletion of the miR-132/212 in PS19 mice, a model of tauopathy, had little effects on disease phenotypes with divergent effects on tau biology. They also discuss potential pitfalls explaining these observations including the lack of adequate tools and animal models to study the involvement of miRNAs in tau pathology.

As evidenced in this Research Topic, because of its key function and its dysregulation and aggregation in Alzheimer's and other tauopathies, tau has become an attractive target for therapy. Existing tau-targeting approaches, their advantages and limitations were elegantly summarized by Masnata et al., with an emphasis on their potential in treating Huntington's disease, a secondary tauopathy. Some approaches target tau phosphorylation using kinase inhibitors, phosphatase activators or immunotherapy targeted at tau phosphorylation at sites that are either hyperphosphorylated or exclusively phosphorylated in Alzheimer's. In the particular case of immunotherapy, a few factors must be considered when designing antibodies including the differential toxicity of tau seeds and the disease-specificity of tau hyperphosphorylation, which precludes the use of immunotherapy targets across tauopathies as discussed by Duquette et al.

The disease-specific characteristics of tau aggregates across tauopathies as revealed by cryo-EM, their trans-cellular propagation and the information gathered from models used to study tau aggregation further prompted the development of approaches targeting tau self-assembly as discussed by Oakley et al. and by Masnata et al. The present most promising avenues include tau aggregation inhibitors, and active or passive immunisation against either post-translational modifications facilitating tau aggregation and conformationally altered forms of tau or aggregated tau, thus preventing the formation of PHFs.

Tau spreading could also be targeted by inhibiting tau receptors favouring spreading. A recent study by Rauch et al. (73) showed that neuronal surface low-density-lipoprotein-receptor-related protein-1 (LRP1) mediates internalization and spreading of both physiological tau and pathogenic tau oligomers, suggesting that LRP1 could be a suitable target for intervention. Through their commentary on the paper by Rauch et al., Fearon and Lynch highlight the potential and limitations of LRP1 as a key player in tau physiology and its potential as a therapeutic target for tauopathies. As previously suggested in Fearon and Lynch's the *in vitro* and animal data do not always translate to human studies.

Other approaches to mitigate the deleterious effects of pathological tau are being examined such as modulating *MAPT* gene expression. As revealed in preclinical studies, the restoration of physiological miRNA levels could also provide an attractive alternative.

As emphasized in several contributions to this Research Topic, models mimicking tau biology in physiological and pathological conditions, while allowing testing potential therapeutics they present considerable limitations. To overcome these limitations Shamir et al. introduce a neuron-like *in vitro* model of human origin which shows the expected toxicity of human-derived PHF-enriched tau and enables studies on the internalization and interaction of tau antibodies with pathological tau.

A significant limitation of mouse tauopathy models is that while mouse models provide invaluable insight into tau biology and pathology, they do not recapitulate the human tau splicing into six isoforms and the human 3R/4R ratio [reviewed in (74, 75)]. There are a few mouse models which mimic human tau pathology leading to brain atrophy such as the PS19 and the P301S tau (76, 77). More recently, P301S tau transgenic mice with targeted replacement of endogenous ApoE with human ApoE revealed the important role of ApoE in regulating tau-mediated neurodegeneration. In this model, the expression of the human ApoE4 protein caused the most severe tauopathy leading to human-like brain atrophy (78). In this regard rat models of tauopathy display significant advantages (74). For example, the McGill-R962-hTau rat model of tauopathy, as similar models under development, overexpresses the longest isoform of human tau (2N4R) with the P301S mutation causative of FTDP-17 under the control of the CaMKII alpha gene promoter. These rats progressively develop human-like tau pathology and related phenotype with tau hyperphosphorylation and conformationally-altered tau, resulting in NFT-like inclusions and neuroinflammation followed by neuronal loss, marked by brain atrophy, ventricular dilation, demyelination, and cognitive impairment (79). Of relevance to the study of the human-like tauopathy and therapeutics rats are genetically and physiologically closer to humans than mice and unlike mice they display all six human tau isoforms, although not at the same ratio. Furthermore, the rat endogenous ApoE protein has a high homology with the human ApoE4 protein.

## Conclusion

This collection of reviews covers a wide variety of progresses regarding the participation of the tau protein in a diversity of neurodegenerative conditions. These contributions bring new ideas regarding physiological and pathological molecular mechanisms involving this multi-faceted microtubule-associated protein. These contributions offer an excellent background to further define the differential pathological aspects of diverse tauopathies. It supports the analysis of tau-mediated glia-neuron interactions and their impact in the homeostatic maintenance of the brain vascular bed. The main future challenge in the field will remain the development of effective therapeutics able to halt or delay the devastating consequences of brain tau pathology. This e-book should be of much assistance to conceive and investigate novel therapeutic approaches of eventual clinical value.

## Author Contributions

SD, MS, and AC have participated in writing the Editorial and approved it for publication. All authors contributed to the article and approved the submitted version.

## Conflict of Interest

The authors declare that the research was conducted in the absence of any commercial or financial relationships that could be construed as a potential conflict of interest.

## Publisher's Note

All claims expressed in this article are solely those of the authors and do not necessarily represent those of their affiliated organizations, or those of the publisher, the editors and the reviewers. Any product that may be evaluated in this article, or claim that may be made by its manufacturer, is not guaranteed or endorsed by the publisher.
